# Repeatability assessment of anterior segment measurements in myopic patients using an anterior segment OCT with placido corneal topography and agreement with a swept-source OCT

**DOI:** 10.1186/s12886-024-03448-z

**Published:** 2024-04-22

**Authors:** Hao Wang, Li-Shuang Zhu, Chen-Jiu Pang, Qi Fan

**Affiliations:** 1 Department of Refractive Surgery, Henan Provincial People’s Hospital, Henan Eye Hospital, People’s Hospital Affiliated to Zhengzhou University, People’s Hospital Affiliated to Henan University, NO.7 Weiwu Road, 450003 Zhengzhou, China; 2 Department of Blood Transfusion, Zhengzhou People’s Hospital, NO.33 Huanghe Road, 450003 Zhengzhou, China

**Keywords:** OCT, Corneal thickness, Keratometry, Corneal volume, Repeatability, Agreement

## Abstract

**Background:**

The precision of anterior segment biometric measurements in eyes has become increasingly important in refractive surgery. The purpose of this study is to assess the repeatability of the automatic measurements provided by a new spectral-domain optical coherence tomograph (SD-OCT)/Placido topographer (MS-39, CSO) and its agreement with a swept-source OCT (SS-OCT) biometer (CASIA SS-1000, Tomey) in patients with myopia.

**Methods:**

The right eye of 235 subjects was scanned 3 times with both devices. The evaluated parameters included central corneal radius of the steep meridian, central corneal radius of the flat meridian, mean central corneal radius, thinnest corneal thickness, central corneal thickness, anterior chamber depth, corneal volume and diameter. The intraobserver repeatability of the MS-39 measurements was calculated using intraclass correlation coefficient (ICC), within subject standard deviation, coefficient of repeatability, coefficient of variation and repeated-measures analysis of variance of the 3 repeated measurements. The agreement between the two devices was evaluated by 95% limits of agreement (LoA).

**Results:**

The majority of the parameters acquired from MS-39 showed high repeatability. The repeatability of corneal diameter was slightly lower than the other measurements, although the ICC remained high. Agreement with the CASIA SS-1000 was good, indicated by the Bland-Altman plots with narrow 95% LoA values for all parameters assessed.

**Conclusions:**

The high repeatability of automatic measurements by the new device supports its clinical application in eyes with myopia, and the good agreement between the two devices indicates they could be used interchangeably for the parameters evaluated.

## Background

Precise assessment of anterior segment parameters is of clinical and investigational importance in multiple fields of ophthalmology. Among them, quantitative evaluation of corneal thickness and keratometry is essential for refractive surgery. The accuracy of these biometric parameters is of critical importance to select the most suitable surgery for every patient and to identify the risk factors of postoperative complications. The development of new surgical techniques for the human eye requires, in many situations, deep understanding of the spatial relationships and dimensions of the anterior segment. Imaging of the anterior segment of the eye has drastically advanced over the past 15 years. A-scan ultrasonography [1, 2] was introduced first, followed by scanning-slit topography [3], examination with a Scheimpflug camera [4] and anterior segment optical coherence tomography (AS-OCT) [5, 6].

AS-OCT devices can produce high-resolution images of the corneal, anterior chamber and the iridocorneal angle [7, 8]. Accurate and precise anterior segment biometry is crucial for the diagnosis and treatment of ocular disorders. Measurement of the central corneal thickness (CCT) helps to reduce the risk of postoperative ectasia when screening for refractive surgery [9] and is crucial in the measurement of intraocular pressure (IOP) [10], and diagnosis of corneal disorders including keratoconus [11]. In refractive surgery, the CCT is decisive measurement for assessing the result and the success of the surgery. AS-OCT is known to produce better images with higher definition, which has made it possible, for example, to measure the corneal epithelial thickness [12], a parameter helping to identify keratoconus, that has never been evaluated by Scheimpflug cameras. The anterior chamber depth (ACD) is an important measurement for screening for primary angle closure glaucoma [13], and performing the optical power calculation of phakic and pseudophakic intraocular lenses (IOLs) [14].

Until now, a greater number of radial scans could be acquired only by the CASIA SS-1000 and subsequently by the CASIA 2 (Casia, Tomey, Nagoya, Japan) systems, both of which use swept-source optical coherence tomography (SS-OCT) and a 1,310 nm light source. These two devices acquire 16 radial B-scans centered on the corneal vertex, each of them 10.0 mm long and 6.0 mm deep, to generate corneal curvature and thickness maps [15]. The anterior segment CASIA SS-1000 OCT (Tomey Corporation, Nagoya, Japan) is a swept-source optical coherence tomography (SS-OCT) device, which produces high-resolution 3D images of the cornea and anterior segment of the eye. This device uses a light source with a wavelength of 1,310 nm and has a high scanning speed of 30,000 A-scans/second, also measuring 256 B-scans over the cornea, which allows real-time 3D image acquisition.

A new SD-OCT device combined with Placido corneal topography, named MS-39 (CSO, Scandicci, FI, Italy), was recently introduced to clinical application [16, 17]. High-resolution OCT and Placido images can be obtained with a single measurement and then reconstructed with faster acquisition times, thus avoiding possible misalignment during the integration of the images obtained from two devices [18].

Validation studies are necessary for any new instrument before it can be adopted in clinical application. It further needs to be reliable in different populations and has the ability to be interchangeably used with other instruments. This study aimed to evaluate the repeatability of anterior segment biometric measurements in myopic patients provided by MS-39, and assess its agreement with the CASIA SS-1000 OCT. As far as we know, this is the first study evaluating the agreement of measurements of anterior segment between MS-39 and CASIA SS-1000.

## Methods

Patients with healthy unoperated corneas, who intended to receive refractive surgery to correct myopia and/or astigmatism, were enrolled in the study. The current study was carried out at the Refractive Surgery Center of Henan Provincial Eye Hospital from April to June in 2021. In Chinese population, most of the patients suffered from refractive errors were myopic, and few of them were hyperopic, so the participants included in our current study were myopic. All the participants had no ocular abnormalities other than refractive error. Approval by the institutional review board of Henan Provincial Eye Hospital [approval code: HNEECKY-2021(06)] was obtained and the study was conducted in accordance with the Declaration of Helsinki. Informed consent was signed by each patient after they were provided a verbal and written explanation of the purpose, type of devices, measurement process details and data treatment of the current study.

The exclusion criteria included the presence of keratoconus or suspicion of keratoconus, previous diagnosis of dry eye, history of corneal disease or trauma, any kind of ocular surgery, poor fixation, using a rigid contact lens within 4 week or soft contact lenes within 2 weeks, systemic diseases and other anterior segment abnormalities. Only the right eye of each patient was investigated due to the similarity of measurements for both eyes [19].

### Instruments

The MS-39 tomographer (software version 3.6) integrates both SD-OCT-based anterior segment tomography and Placido-disk corneal topography modalities into one device to acquire measurements of the anterior segment of the eye. The 840 nm superluminescent LED light source enables MS-39 to acquire images of the total cornea at a higher resolution than the Scheimpflug camera, namely with an axial resolution of 3.5 nm, a transverse resolution of 35 nm, and a maximum depth of 7.5 mm. Moreover, it can scan 25 meridians on a 16-mm transversal field, and measure 31,232 and 25,600 points of the cornea in the anterior and posterior surfaces, respectively. Following autocalibration, the scanning process simultaneously obtains (in about 1 s) one keratoscopy, one iris front image (for the detection of pupil) as well as a sequence of 25 SD-OCT radial scans at a wavelength of 840 nm, to image the anterior segment, including the anterior cornea, posterior cornea, anterior lens and iris [17, 18].

The CASIA SS-1000 is an AS-OCT using 1,310 nm high-speed swept-source OCT technology. Images are generated using a rate of 30,000 A-scans per second, with an axial resolution less than 10 μm and a transverse spatial resolution less than 30 μm. The dimensions of large depth scan are 16 mm vertical by 16 mm horizontal, with a 6 mm tissue penetration. Sixteen radial B scans and 512 A scans can be obtained in 0.3 s, thus building a 3D image in the corneal map mode. The Topo-Pachy-Map scan protocol also takes 0.3 s to acquire evenly spaced 16 radial B scans. The map calculates and provides the corneal topographic and thickness data of the anterior and posterior corneal surfaces [20].

### Measurements

A complete ophthalmic examination was performed on all individuals, using instruments according to the guidelines of the manufacturer. Both devices were calibrated and the eyes were scanned three consecutive times on each device by the same experienced examiner (Q.F.), in a random order generated from MedCalc software version 15.2.2 for Windows (MedCalc software Ltd, Ostend, Belgium). Each subject was appropriately positioned on the chin rest and with the forehead resting on the device, and the eye was aligned with a central fixation light. The patients were instructed to fully blink just before each measurement to gain a smooth tear film. All measurements were performed in a dark room between 9:00 am and 5:00 pm to reduce the effects from diurnal ocular changes. Scans were taken in the automatic release mode. Only qualified scans indicated by the instrument were used for analysis; otherwise, the measurements were repeated. The entire procedure lasted less than 30 min.

The obtained anterior segments topographies for MS-39 were managed by the systematic software (version 3.6), which performed biometric measurements with the built-in tools. The following biometric measurements were obtained from the MS-39 images: central corneal radius of the steep meridian (K_s_), central corneal radius of the flat meridian (K_f_), mean central corneal radius (K_m_), thinnest corneal thickness (TCT), CCT, ACD, corneal volume (CV) and corneal diameter (CD). The keratometry mentioned above was defined as the average corneal power calculated at a 3 mm ring, based on anterior corneal axial curvature. The conversion of anterior radius to keratometry values is performed using the standard keratometric index of 1.3375. Additionally, the built-in software in the CASIA SS-1000 automatically calculated the K_s_, K_f_, K_m_, TCT and CCT.

### Statistical analysis

Statistical processing of data was conducted using SPSS (version 25.0) for Windows (IBM Corp., Armonk, NY, USA) and MedCalc (version 15.2.2) for Windows. The data were reported as mean ± standard deviation (SD). The normality of the obtained data was assessed using the Kolmogorov-Smirnov test, in order to be able to use parametric tests. All association tests were considered to be statistically significant, if the *P* value was less than 0.05.

Only data from the right eye of each subject was analyzed to avoid intra-subject codependence. The intraobserver repeatability of the 3 repeated measurements for MS-39 was evaluated using the following parameters: intraclass correlation coefficient (ICC), within subject standard deviation (S_w_), coefficient of repeatability (CoR) and coefficient of variation (CoV). ICC is defined as the ratio of the between-subject variance to the sum of the pooled between-subject variance and the within-subject variance. Repeatability was classified as high level if the ICC was at least 0.8. S_w_ is defined as the square root of the mean variance between subjects. CoR, also known as limits of repeatability or intrasession test-retest variability, was calculated as 1.96$\sqrt{2}$×*S*_*w*_ [21]. CoV was calculated as S_w_ divided by the average value, shown as CoV = $\frac{Sw}{\stackrel{-}{x}}$, and expressed as a percentage. Additionally, repeated-measures analysis of variance (ANOVA) with *post hoc* Bonferroni test was utilized to estimate the repeatability of the measurements mentioned above. The repeatability of MS-39 was also evaluated by Bland-Altman analysis using MedCalc, through comparison among the three repeated measurements. The 95% limits of agreement (LoA) were calculated as the mean differences among two repeated measurements ± 1.96 × SD of the differences.

The average of three repeated measurements from each device was used to evaluate the agreement between MS-39 and CASIA SS-1000. The agreement between the two systems was graphically assessed by a Bland-Altman plot and numerically by the 95% LoA. Graphs were created reflecting the difference between the two devices plotted against the mean values of the two devices [22]. Furthermore, differences between the mean values measured by the two devices were evaluated by a paired *t*-test.

## Results

### Characteristics of participants

In total 235 patients had their right eye examined in the current study. All patients did not suffer from any ocular abnormalities other than refractive error. The mean age of patients was 25.97 ± 5.78 years (ranging from 18 to 40 years, 107 males and 128 females). The mean spherical equivalent of the participants was − 5.63 ± 2.07 diopters (D) (ranging from − 10.75 to -1.5D), while the mean axial length was 25.28 ± 1.34 mm. All data obtained followed the normal distribution, as evaluated by the Kolmogorov-Smirnov test. Table 1 shows the means, medians and 95% confidence intervals (CI) of the parameters analyzed in this study.

**Table 1:** Means, medians and 95%CIs for the measurements of each examined parameter from MS-39 and CASIA SS-1000

Parameter	MS-39				CASIA SS-1000
	Mean ± SD (SE)	95%CI		Mean ± SD (SE)	95%CI
K_f_ (D)	42.90 ± 1.19 (0.08)	[42.81, 42.99]		42.87 ± 1.18 (0.08)	[42.72, 43.02]
K_s_ (D)	44.06 ± 1.34 (0.09)	[43.96, 44.16]		44.05 ± 1.34 (0.09)	[43.88, 44.22]
K_m_ (D)	43.47 ± 1.21 (0.08)	[43.38, 43.56]		43.44 ± 1.22 (0.08)	[43.29, 43.60]
TCT (µm)	531.85 ± 34.08 (2.22)	[529.33, 534.36]		526.80 ± 33.66 (2.19)	[522.47, 531.12]
CCT (µm)	535.99 ± 34.17 (2.23)	[533.47, 538.52]		531.82 ± 34.06 (2.21)	[527.45, 536.20]
ACD (mm)	3.27 ± 0.24 (0.01)	[3.26, 3.29]		NA	NA
CV (mm^3^)	57.69 ± 3.30 (0.12)	[57.45, 57.94]		NA	NA
CD (mm)	11.87 ± 0.38 (0.01)	[11.84, 11.89]		NA	NA

CI = confidence interval; K_f_ = central corneal radius of the flat meridian; K_s_ = central corneal radius of the steep meridian; K_m_ = mean central corneal radius; TCT = thinnest corneal thickness; CCT = central corneal thickness; ACD = anterior chamber depth; CV = corneal volume; CD = corneal diameter; NA = not available

### Repeatability

Table 2 shows the ICC, S_w_, CoR, CoV values and the results of repeated-measures ANOVA for the parameters measured by MS-39. MS-39 presented excellent repeatability, with an ICC > 0.95 in all measurements. The repeatability for CD was slightly lower than that of the other parameters, although the ICC remained high. The S_w_ of MS-39 in measuring K_s_, K_f_, K_m_, TCT, CCT, ACD, CV and CD was 0.079D, 0.091D, 0.067D, 0.880 μm, 0.700 μm, 0.014 mm, 0.183mm^3^, 0.066 mm, respectively, while the CoR of MS-39 in measuring K_s_, K_f_, K_m_, TCT, CCT, ACD, CV and CD was 0.219D, 0.252D, 0.186D, 2.438 μm, 1.939 μm, 0.039 mm, 0.507mm^3^, 0.183 mm, respectively. The CoV value obtained for all examined parameters was less than 1%, indicating the great repeatability of MS-39. No statistical significance was detected in any result of repeated-measures ANOVA for the measurements of K_s_, K_f_, K_m_, TCT, CCT, ACD, CV or CD (*P* > 0.05 in all analyses). Furthermore, Bonferroni correction was performed to compare the differences of every two of the three repeated measurements for the examined parameters from MS-39 (Table 3). The difference was not statistically significant in any of the *post hoc* Bonferroni tests (*P* > 0.05 in all the tests). The results of Bland-Altman analysis showed that each repeated measurement presented a narrow 95% LoA with the other two, which indicates good repeatability.

**Table 2:** Repeatability analysis of biometric measurements provided by MS-39

Parameter	ICC (95%CI)	S_w_	CoR	CoV (%)	Repeated-measures ANOVA	
					F	*P*
K_f_ (D)	0.9956(0.9945 to 0.9965)	0.079	0.219	0.184	0.82	0.440
K_s_ (D)	0.9954(0.9942 to 0.9963)	0.091	0.252	0.207	1.36	0.258
K_m_ (D)	0.9970(0.9963 to 0.9976)	0.067	0.186	0.154	0.12	0.889
TCT (µm)	0.9993(0.9992 to 0.9995)	0.880	2.438	0.165	1.66	0.190
CCT (µm)	0.9996(0.9995 to 0.9997)	0.700	1.939	0.131	2.63	0.073
ACD (mm)	0.9988(0.9984 to 0.9990)	0.014	0.039	0.428	2.03	0.132
CV (mm^3^)	0.9970(0.9962 to 0.9976)	0.183	0.507	0.317	2.34	0.098
CD (mm)	0.9693(0.9620 to 0.9754)	0.066	0.183	0.556	0.50	0.608

ICC = intraclass correlation coefficient; CI = confidence interval; S_w_ = within subject standard deviation; CoR = coefficient of repeatability; CoV = coefficient of variation; ANOVA = analysis of variance; K_f_ = central corneal radius of the flat meridian; K_s_ = central corneal radius of the steep meridian; K_m_ = mean central corneal radius; TCT = thinnest corneal thickness; CCT = central corneal thickness; ACD = anterior chamer depth; CV = corneal volume; CD = corneal diameter

**Table 3:** Repeatability analysis of biometric measurements provided by MS-39

8Parameter	Bonferroni whirection (*P*)				Bland-Altman [mean difference (95%LoA)]		
	1st vs. 2nd	1st vs. 3rd	2nd vs. 3rd		1st vs. 2nd	1st vs. 3rd	2nd vs. 3rd
K_f_ (D)	0.5675	1.0000	1.0000		0.01(-0.20 to 0.22)	0.00(-022 to 0.22)	-0.01(-0.23 to 0.22)
K_s_ (D)	0.1891	0.9929	1.0000		-0.01(-0.23 to 0.21)	-0.01(-0.29 to 0.27)	0.00(-0.25 to 0.26)
K_m_ (D)	1.0000	1.0000	1.0000		0.00(-0.17 to 0.17)	0.00(-0.20 to 0.19)	0.00(-0.19 to 0.19)
TCT (µm)	0.1075	1.0000	1.0000		-0.1(-2.3 to 2.0)	-0.1(-2.8 to 2.6)	0.1(-2.4 to 2.6)
CCT (µm)	0.7150	0.0972	0.6944		-0.1(-1.7 to 1.6)	-0.1(-2.2 to 1.9)	-0.1(-2.1 to 2.0)
ACD (mm)	0.9098	0.1404	0.9675		0.00(-0.02 to 0.02)	0.00(-0.02 to 0.03)	0.00(-0.02 to 0.02)
CV (mm^3^)	1.0000	0.1510	0.1722		-0.01(-0.57 to 0.56)	-0.04(-0.57 to 0.50)	-0.03(-0.43 to 0.38)
CD (mm)	1.0000	1.0000	1.0000		0.00(-0.12 to 0.12)	-0.01(-0.23 to 0.22)	0.00(-0.19 to 0.19)

LoA = limits of agreement; K_f_ = central corneal radius of the flat meridian; K_s_ = central corneal radius of the steep meridian; K_m_ = mean central corneal radius; TCT = thinnest corneal thickness; CCT = central corneal thickness; ACD = anterior chamer depth; CV = corneal volume; CD = corneal diameter

### Agreement

**Fig. 1:**
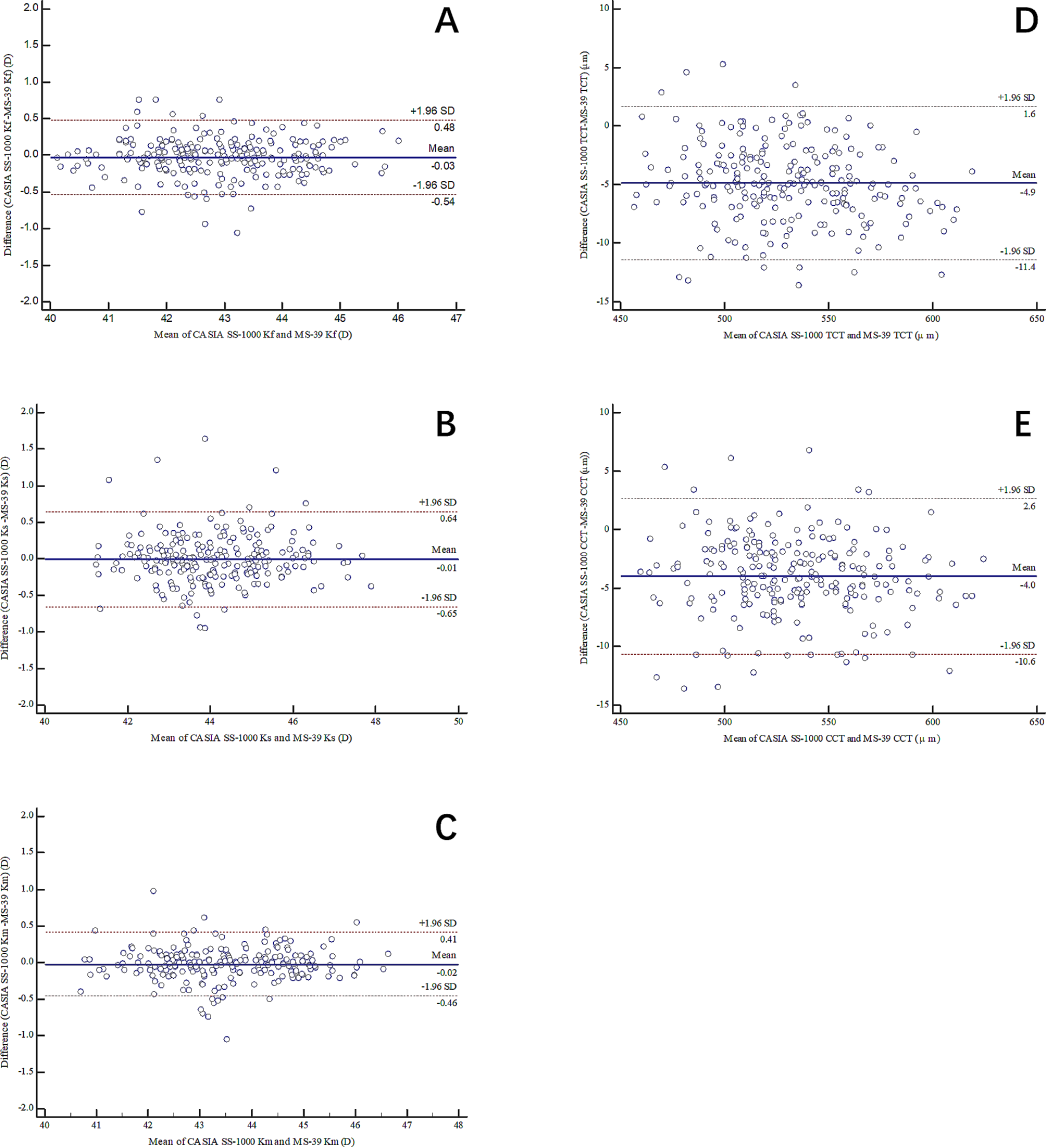
Bland-Altman plots comparing the anterior segment parameters between MS-39 and CASIA SS-1000 Bland-Altman plots comparing the values of (**a**) central corneal radius of the flat meridian (K_f_), (**b**) central corneal radius of the steep meridian (K_s_), (**c**) mean central corneal (K_m_), (**d**) thinnest corneal thickness (TCT), and (**e**) central corneal thickness (CCT) obtained by the MS-39 (CSO) and CASIA SS-1000 (Tomey) (The solid line in the middle represents the mean difference, and the two dotted lines above and below represent the 95% limits of agreement (LoA)).

The differences of the measurements between the two devices are shown in Table 4. No statistically significant differences were observed in the K_f,_, K_s_ and K_m_ measurements. Statistically, but not clinically significant differences were observed in the measurements of TCT and CCT. The CASIS SS-1000 underestimated the TCT and CCT values compared with MS-39 (both *P* < 0.0001). The 95% LoA values of each parameter are displayed in Table 4; Fig. 1. The 95% LoA values for K_f_, K_s_, K_m_, TCT and CCT were all narrow, implying good agreement between the two devices and clinically irrelevant differences.

**Table 4:** Agreement between MS-39 and CASIA SS-1000 in biometric measurements

Parameter	Mean difference ± SD	*P*	95% LoA
K_f_ (D)	-0.029 ± 0.260	0.086	-0.54 to 0.48
K_s_ (D)	-0.007 ± 0.330	0.749	-0.65 to 0.64
K_m_ (D)	-0.024 ± 0.221	0.102	-0.46 to 0.41
TCT (µm)	-4.893 ± 3.331	< 0.0001	-11.4 to 1.6
CCT (µm)	-4.001 ± 3.385	< 0.0001	-10.6 to 2.6

SD = standard deviation; LoA = limits of agreement; K_f_ = central corneal radius of the flat meridian; K_s_ = central corneal radius of the steep meridian; K_m_ = mean central corneal radius; TCT = thinnest corneal thickness; CCT = central corneal thickness

## Discussion

Ocular anterior segment topography devices have been rapidly developed in recent years, since the interest in corneal refractive surgery and refractive phakic intraocular lens implantation has significantly increased. A new diagnostic device must produce readings with good repeatability in order to acquire widespread application. The current study was performed to assess the repeatability of anterior segment measurements provided by MS-39, a new AS-OCT device combined with Placido corneal topography. The present study revealed that all obtained parameters, including K_s_, K_f_, K_m_, TCT, CCT, ACD, CV and CD, were highly repeatable with MS-39.

The repeatability of automatic measurements by MS-39 was evaluated in a previous study reported by Schiano-Lomoriello et al. [23]. The study enrolled 96 participants and reported that MS-39 could provide repeatable measurements of simulated keratometry, total corneal power, TCT, aqueous depth and CD, which is in agreement with the results from this study for the common parameters assessed. The present study assessed a larger group of participants, and the repeatability of measurement of CV with MS-39 was also evaluated, with congruent results, which were not included in the previous report from Schiano-Lomoriello et al. [23].

With reference to keratometric measurements, the repeatability of K_s_, K_f_ and K_m_ with MS-39 of this study was shown to be excellent. A previous study by Chan et al. [24] showed that the repeatability of K_s_, K_f_ and K_m_ acquired from CASIA SS-1000 and Pentacam HR rotating Scheimpflug camera (Oculus Inc., Lynnwood, WA, US) was comparable to the current study. Biswas et al. [25] compared the repeatability of keratometric measurements with CASIA SS-1000, Pentacam HR and Topographic Modeling System, version 5 (TMS-5, Tomey Corp., Nagoya, Japan), and showed that the CASIA SS-1000 and Pentacam HR presented similar repeatability with this study, while TMS-5 showed relatively inferior results. Only the repeatability of K_m_ was evaluated in the study from Panthier et al., who reported that IOL Master 700 (Carl Zeiss, Germany) and Anterion (Heidelberg Engineering GmbH, Heidelberg, Germany), which both belong to SS-OCT, showed good repeatability when measuring K_m_, the result of which was comparable to the current study.

Corneal thickness is one of the parameters used to determine the suitability of a patient for any corneal refractive surgery, or further applications like keratoconus detection or orthokeratology procedures. Previous studies [23–25] on healthy eyes obtained average values of TCT between 530 and 550 μm with AS-OCT, which are comparable to the reported average of 531.85 ± 34.08 μm in this study. The repeatability of TCT measurements with MS-39 was improved compared with the reported CASIA SS-1000 measurements in the literature [24, 25]. Savini et al. [17] evaluated the repeatability of TCT measurements with MS-39, acquiring a CoR of 4.77 μm, a CoV of 0.32%, and an ICC of 0.999. Similar results were obtained in the current study, where CoR was 2.44 μm, CoV was 0.17%, and ICC was 0.999. In terms of CCT measurement, excellent repeatability was confirmed by the small COR (1.94 μm) and CoV (0.131%) scores and the ICC of 0.9996 from the current study. Measurements of CCT from MS-39 produced mean values (535.99 ± 34.17 μm) comparable to those previously reported using SS-OCT on healthy eyes [24–29]. Previous studies on myopic eyes showed that Pentacam HR rotating Scheimpflug camera and Sirius Scheimpflug camera-Placido topographer (CSO, Scandicci, FI, Italy) achieved inferior repeatability, based on the results from the previous studies that the ICC was no more than 0.998, 0.992, 0.997, 0.985, 0.977 for CCT, TCT, ACD, Kf and Ks, respectively [30–32].

In the current study, ACD was calculated as the distance between the endothelium and the anterior lens surface, according to the definition by Hoffer [33], to remain consistent with the literature. High repeatability was also acquired in ACD measurements from MS-39. The repeatability of ACD was similar with that reported by Schiano-Lomoriello et al. [23], although the ACD acquired in this study was slightly deeper, potentially due to the different population enrolled. Moreover, the new SD-OCT/Placido topographer showed higher repeatability in ACD measurements than those obtained from Scheimpflug cameras [30] and SS-OCT [28].

In terms of CD measurement, good repeatability was confirmed by the small CoR (0.183 mm) and CoV (0.556%) scores, as well as the ICC of 0.9693. Accurate CD assessment is critical for phakic intraocular lens implantation to minimize vault-related problems like cataract and glaucoma. A previous study in eyes with myopia showed that the IOL Master 700 achieved relatively inferior repeatability of CD measurements compared to the current study [26], but the study from Schiano-Lomoriello et al. showed a comparable result [23].

The repeatability with MS-39 in terms of CV measurements was shown to be excellent, obtaining a CoR of 0.507 mm^3^, a CoV of 0.32%, and an ICC of 0.997. To the knowledge of the authors, this is the first study evaluating the repeatability of CV with MS-39. No previous studies have focused on the repeatability of CV measurements by AS-OCT in myopic patients. The stability of CV is essential for patients after refractive surgery, especially for small incision lenticule extraction [34].

The good repeatability of the anterior segment measurements from MS-39 shown by the current study, reflected the reliability of MS-39 in the field of ophthalmology, especially for patients intended to receive refractive surgery. MS-39 could use elevation maps of the anterior and posterior corneal surface, and pachymetric maps to screen keratoconus before surgery, and could be a warning sign for postoperative ectasia in the follow-up stage.

The agreement of the biometric measurements between the MS-39 SD-OCT/Placido topographer and CASIA SS-1000 SS-OCT was also investigated, and all parameters were found to be clinically interchangeable. To the knowledge of the authors, this is the first study to compare the two devices. In a previous study, agreement with the measurements of the MS-39 SD-OCT/Placido topographer and Pentacam HR rotating Scheimpflug camera/Sirius Scheimpflug camera-Placido topographer was high for ACD and TCT in myopic patients [17]. The MS-39 SD-OCT/Placido topographer was compared with the Argos SS-OCT biometer (Movu Inc., CA, US) in another study, and high agreement was reported between the two devices in measuring CCT, ACD, K_m_ and astigmatism [18].

There are advantages as well as limitations in this study, compared with previous studies. The most important advantage is the large population examined, which included 235 eyes from 235 myopic patients, considerably more than previous studies [16, 22–27, 34–37]. Additionally, only the right eye from each participant was measured, which eliminated the bias generated from bilateral-eye data. On the other hand, there are two limitations meriting consideration. First, the subjects enrolled in the current study were all myopic patients, whereas no pathologic eye, such as keratoconus or cataract, was included. Second, only the commonly used parameters from the two devices were investigated. Future research will focus on the investigation of the remaining parameters. Thirdly, the epithelial thickness, which could be obtained by the two instruments, was not compared in the current study. We will try to include the investigation of epithelial thickness in our further studies.

## Conclusions

In conclusion, highly repeatable measurements of anterior segment parameters, including K_s_, K_f_, K_m_, CCT, TCT, ACD, CV and CD, were achieved in individuals with myopia using MS-39, the new SD-OCT/Placido topographer. Good agreement was found between MS-39 and CASIA SS-1000, indicating both devices can be applied interchangeably for the parameters assessed. The results of the current study indicate MS-39 to be a reliable device for ophthalmologists in the preoperative examinations before refractive surgery and the follow up for the patients after surgery in the future.

## Data Availability

The datasets used and/or analyzed during the current study are available from the corresponding author on reasonable request.

## Abbreviations

AS-OCT: anterior segment optical coherence tomography
CCT: central corneal thickness
IOP: intraocular pressure
ACD: anterior chamber depth
IOL: intraocular lens
SD-OCT: spectral-domain optical coherence tomography
SS-OCT: swept-source optical coherence tomography
K_s_: central corneal radius of the steep meridian
K_f_: central corneal radius of the flat meridian
K_m_: mean central corneal radius
TCT: thinnest corneal thickness
CV: corneal volume
CD: corneal diameter
SD: standard deviation
ICC: intraclass correlation coefficient
S_w_: within subject standard deviation
CoR: coefficient of repeatability
CoV: coefficient of variation
ANOVA: analysis of variance
LoA: limits of agreement
D: diopter
CI: confidence intervals
